# Pollen grain morphology is not exclusively responsible for pollen collectability in bumble bees

**DOI:** 10.1038/s41598-019-41262-6

**Published:** 2019-03-18

**Authors:** Sabine Konzmann, Sebastian Koethe, Klaus Lunau

**Affiliations:** Institute of Sensory Ecology, Heinrich Heine University Düsseldorf, Düsseldorf, Germany

## Abstract

Bee-pollinated plants face a dilemma in that bees both passively transport pollen grains among conspecific flowers and actively collect pollen to feed their larvae. Therefore, mechanisms that reduce pollen collection by bees have evolved in melittophilous plants. Malvaceae pollen is uncollectable for corbiculate bees which has previously been ascribed to pollen size, spines, and pollenkitt. We analysed the influence of pollen grain properties (diameter, spine length, spine density) on the collectability of echinate (spiny) pollen by bumble bees (*Bombus terrestris*). Workers individually foraging on one of eight plant species from six families performed significantly less pollen foraging on plants which have large, echinate pollen grains. Nevertheless, neither pollen grain size, spine length, nor spine density prove to be an absolute disqualifier for collectability. While pollen foragers did not shift to nectar collection but seized visiting flowers with uncollectable pollen, nectar foragers performed regular foraging bouts on these plants. Pollen that is uncollectable for corbiculate bees limits pollen depletion by generalist bumble bees and probably also honey bees while maintaining them as pollinators, which is an effective solution to the pollen dilemma. As previous assumptions about the impact of pollen morphology on its collectability are disproved, potentially determining factors are discussed.

## Introduction

Bees collect pollen as a protein source that is crucial for egg maturation^1^ and larval development^2–4^. Although many bees are efficient pollinators of a broad range of plant species, only a small fraction of the pollen adhering to a bee’s body contributes to pollination^5–8^. For instance, in *Campanula rapunculus* (Campanulaceae) only 3.7% of the pollen grains of one flower are transferred for pollination, although bees collect 95.5% of the pollen and leave 0.8% in the flower^9^. Despite legitimate pollinator activity, pollen is lost from pollination because of a variety of circumstances; for example, pollen foragers lose some pollen in the process of packing it and nectar foragers can actively remove accidentally acquired pollen grains by grooming. An arguably large proportion of pollen loss is caused by flower visitors frequenting different plant species during a foraging bout, thus leading to pollen being transferred to stigmata of flowers belonging to heterospecific plants or to non-fertile structures, and covering of the stigma with foreign pollen^10–15^. Pollen collected by corbiculate bees is agglutinated with regurgitated nectar, thus increasing adhesion between pollen grains^16–19^, but diminishing their viability and availability for stigmatic pollen deposition^20^.

The conflict of interest between a plant’s production of pollen for the purpose of reproduction and efficient pollen collection by bees to provide protein for their larvae, the “pollen dilemma”, underlies many plant-pollinator adaptations^21,22^. Several protective mechanisms that decrease pollen loss elicited by flower visitors and increase the chance of pollination have evolved. For instance, flowers of the family Fabaceae hide their pollen from sight with transformed petals, and flowers of the family Lamiaceae feature a staminal lever mechanism to transfer pollen to safe sites on a bee’s body that are difficult to groom or cannot be groomed at all^23–25^. Another protective measure is the echinate pollen grain structure of plants in various families^26,27^, which we examined in this study.

The generative and vegetative cells of pollen grains are coated with two layers: the inner intine, a thin layer composed of cellulose fibrils, and the outer exine, consisting largely of sporopollenin^28–30^. The sporopollenin forms the exterior structure of the pollen grain, producing, for example, an echinate (spiny), psilate (smooth), or reticulate (netlike) surface. The adhesion of pollen grains to flower visitors positively affects the transport distance and is ensured by both pollenkitt and echinate surface structures^31,32^, but also aided by non-morphological features such as electrical charge^33^.

Previous laboratory tests demonstrated that pollen of *Alcea rosea* (Malvaceae) is mechanically protected from being compacted into the corbiculae of bumble bees by its long spines and the pollenkitt covering the grains^27^. When spines were bent by vortexing or pollenkitt was removed by washing, workers of the buff-tailed bumble bee (*Bombus terrestris*, Apidae) could collect the pollen grains. This indicates that – independently of one another – pollenkitt and long spines on the pollen grain surface of *A*. *rosea* hinder pollen collection by bees^27^. We sought to define pollen grain properties determining collectability for bumble bees by testing plants from different families under more natural conditions. We selected six plant species that differed in their pollen grain diameter, spine length, and density of spines – as these morphological characteristics are likely to physically impede pollen packing – to identify the cause of the bees’ inability to pack echinate pollen into their corbiculae. We hypothesize that either one morphological trait or a combination of more than one trait (e.g. grain size and spine length) determines collectability of echinate pollen by bumble bees. To assess pollen collectability, we measured the number of visited flowers, the time that bumble bees spent collecting pollen, and the amount of pollen they gathered in their corbiculae in one foraging bout. We observed whether they cease to visit flowers with uncollectable pollen or if they shift to collecting nectar instead. Additionally, we compared the foraging behaviour of pollen- and nectar-collecting bumble bees on *Malva sylvestris* (Malvaceae).

## Materials and Methods

### General conditions

All tests were conducted in an outdoor flight cage, consisting of an aluminium frame covered with net fabric, in the Botanical Garden of the Heinrich Heine University Düsseldorf, Germany. A colony of *Bombus terrestris* (Biofa AG, Münsingen, Germany) was maintained in this flight cage and provided with diverse flowering plants from various families. Additionally, a feeder containing a 30% sugar solution provided a carbohydrate supply to the colony. To motivate bees to collect pollen from the plants in the flight cage, supplemental pollen was not provided. Most bumble bees collected both nectar and pollen, but during one foraging bout, they settled on one resource. During a given bout, foragers either visited flowers to collect nectar or they collected mainly pollen and also some nectar to pack the pollen grains in their corbiculae. According to their bout-based foraging task, we defined the bumble bees as nectar and pollen foragers for individual trials.

### Pollen characteristics

**Figure 1:**
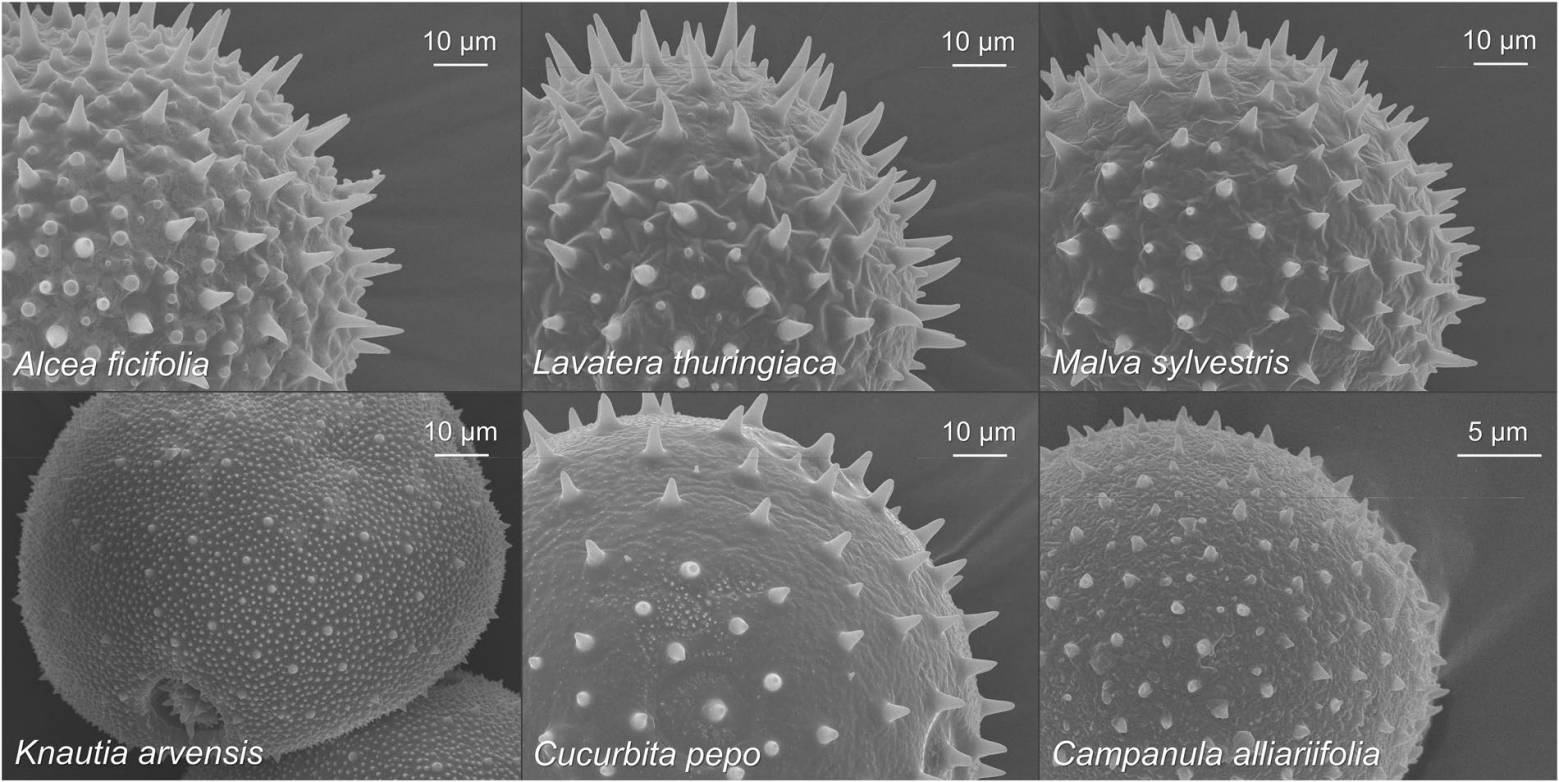
SEM micrographs of fresh echinate pollen grains from *A*. *ficifolia*, *L*. *thuringiaca*, *M*. *sylvestris* (Malvaceae), *K*. *arvensis* (Dipsacaceae), *C*. *pepo* (Cucurbitaceae), and *C*. *alliariifolia* (Campanulaceae) tested in the collectability experiment.

To determine whether specific pollen grain characteristics of echinate pollen influence foraging behaviour of pollen-collecting bumble bees, we surveyed pollen collection from eight plant species. Six species in four families have echinate pollen grains: *Alcea ficifolia*, *Lavatera thuringiaca*, and *Malva sylvestris* (Malvaceae); *Knautia arvensis* (Dipsacaceae), *Cucurbita pepo* (Cucurbitaceae), and *Campanula alliariifolia* (Campanulaceae). Two species have non-echinate pollen: *Verbascum phlomoides* (Scrophulariaceae) and *Rosa arvensis* (Rosaceae). SEM micrographs of pollen samples from the six plant species with echinate pollen grains (Fig. 1) were obtained in the Centre for Advanced Imaging (CAi) of the Heinrich Heine University Düsseldorf. Images of the two non-echinate species were taken with a light microscope. All micrographs were analysed to quantify mean pollen grain diameter (n = 10) and mean spine length (n = 50) with the program ZEN 2012, blue edition (Carl Zeiss Microscopy GmbH, Jena, Germany). To extrapolate the total number of spines, we chose a circle with a diameter equal to the pollen grain radius, then counted the spines within the circle and multiplied that number by eight. Finally, spine density (n μm^−2^) was estimated by dividing spine number (n) by the pollen grain surface area (= 4πr^2^; assuming that the pollen grain is a perfect sphere). These pollen grain properties (Table 1) were compared to their collectability by bumble bees.

**Table 1:** Pollen grain properties (mean ± SD) of eight tested plant species. Exine sculpturing is reticulate for *Verbascum phlomoides* and psilate for *Rosa arvensis*.

Plant species	Diameter [µm]	Spine length [µm]	Spine number [n]	Spine density [n µm^−2^]
Malvaceae				
*Alcea ficifolia*	134.10 ± 5.22	10.18 ± 1.39	210.67 ± 38.55	0.0024 ± 0.0001
*Lavatera thuringiaca*	111.03 ± 2.95	8.80 ± 1.12	126.67 ± 8.54	0.0033 ± 0.0004
*Malva sylvestris*	104.17 ± 6.94	6.77 ± 0.98	133.33 ± 5.96	0.0066 ± 0.0008
Dipsacaceae				
*Knautia arvensis*	99.77 ± 3.47	1.55 ± 0.35	130.67 ± 23.40	0.0041 ± 0.0007
Cucurbitaceae				
*Cucurbita pepo*	139.42 ± 15.41	5.98 ± 0.96	114.67 ± 25.16	0.0018 ± 0.0005
Campanulaceae				
*Campanula alliariifolia*	34.01 ± 3.16	0.96 ± 0.12	184.00 ± 24.87	0.0517 ± 0.0109
Scrophulariaceae				
*Verbascum phlomoides*	22.48 ± 1.02	—	—	—
Rosaceae				
*Rosa arvensis*	29.58 ± 1.68	—	—	—

### Pollen collectability experiment

Prior to testing, we covered plants with mosquito nets to ensure that the bumble bees were unexperienced with these flowers. For each test, one forager that had just left the nest was admitted in the net covering the plants of a selected species. The number of open flowers and amount of available resources enabled each worker to forage *ad libitum*. If a bee did not forage at all or solely drank nectar, it was captured and marked with a numbered opalith tag (Heinrich Holtermann KG, Brockel, Germany). If a forager started collecting pollen, we measured the number of flowers (or inflorescences) visited and the handling time, which was measured with a stopwatch and defined as the time spent collecting pollen from flowers, grooming, and packing the pollen into the corbiculae or discarding the pollen (stationary on flowers and during flight). Preliminary tests demonstrated that handling time seems to be a suitable indicator of pollen collectability: brief handling (<50 s) indicates unsuccessful, aborted pollen collection, medium handling (~ 50–250 s) suggests successful pollen collection, whereas long handling (>250 s) hints at more challenging pollen collection. After a bumble bee ceased visiting flowers, it was captured and marked, and if it had gathered pollen, the pollen load on one hind leg was removed to determine the fresh mass of the collected pollen. Marked foragers were not tested again on a particular plant species, but some were tested later on other plant species.

Owing to heat and periods of rain, the number of workers tested per plant species varied between 5 (*C*. *alliariifolia*, *V*. *phlomoides*, and *R*. *arvensis*) and 10 (*A*. *ficifolia*, *L*. *thuringiaca*, *M*. *sylvestris*, *K*. *arvensis*, and *C*. *pepo*). To assess whether nectar-foraging workers were also influenced by pollen collectability, we recorded the number of flowers visited and the handling time for 10 nectar-collecting bees on *M*. *sylvestris*.

### Statistical Methods

All data were analysed using R statistical software, version 3.5.1^34^. After testing normality of the data with the Shapiro-Wilk test, Kruskal-Wallis test was used for analyses of multiple sets of non-parametric data with Mann-Whitney *U* test including fdr correction as *post hoc* analysis. Mann-Whitney *U* test (non-parametric data) or Student’s *t*-test (parametric data) were used for analyses between two sets of data.

## Results

### Pollen collectability experiment

**Figure 2:**
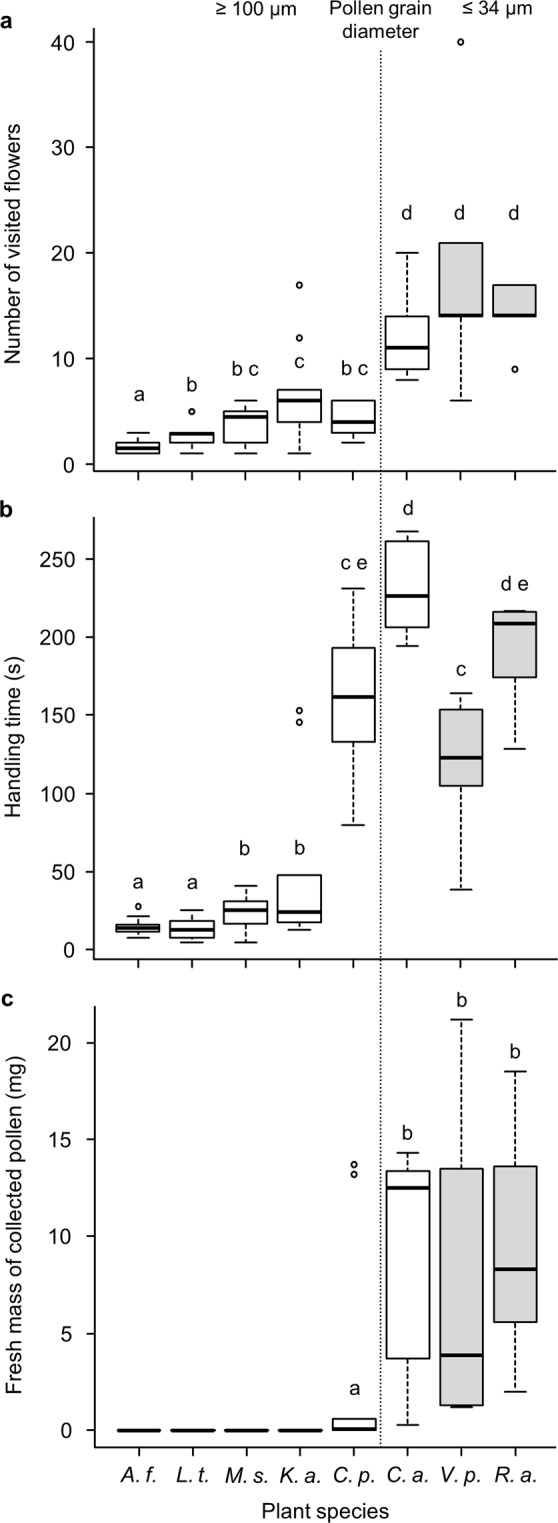
Pollen collection by individual *Bombus terrestris* workers. (**a**) Number of visited flowers, (**b**) handling time, and **(c)** fresh mass of pollen collected during one foraging bout. Plant species are categorized by mean pollen grain diameter (left of the vertical, dotted line = 100–139 µm; right = 23–34 µm) and colour-coded for their exine structure (white = echinate; grey = non-echinate). Ten bees sampled for *Alcea ficifolia*, *Lavatera thuringiaca*, *Malva sylvestris*, *Knautia arvensis*, and *Cucurbita pepo*, and 5 bees sampled for *Campanula alliariifolia*, *Verbascum phlomoides*, and *Rosa arvensis*. Different letters represent significant differences after Kruskal-Wallis test and Mann-Whitney *U* test with fdr correction (*P* < 0.05).

The number of visited flowers and handling time during a foraging bout differed significantly between the tested plant species (Fig. 2a,b; Kruskal-Wallis test: χ^2^ = 44.488, df = 7, *P* < 0.0001 and χ^2^ = 50.074, df = 7, *P* < 0.0001). Pollen-collecting bumble bees visited significantly more flowers of species with small pollen grains (23–34 μm) than flowers of plant species with large pollen grains (100–139 μm). The recorded handling time for all three Malvaceae species and *K*. *arvensis* was significantly shorter than for the other four plant species. Accordingly, the success of pollen collection – measured by the mean mass of collected pollen – also differed significantly between the tested plant species (Fig. 2c; Kruskal-Wallis test: χ^2^ = 51.897, df = 7, *P* < 0.0001). Bees could not collect pollen from any Malvaceae species or *K*. *arvensis*, and only four out of ten foragers collected pollen from *C*. *pepo* flowers – with widely varying success (either <1 or >13 mg fresh mass).

### Effect of pollen grain characteristics

**Figure 3:**
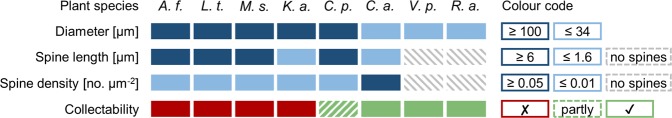
Comparison of pollen grain characteristics and pollen collection by *Bombus terrestris* workers in eight tested plant species: *Alcea ficifolia*, *Lavatera thuringiaca*, *Malva sylvestris* (Malvaceae), *Knautia arvensis* (Dipsacaceae), *Cucurbita pepo* (Cucurbitaceae), *Campanula alliariifolia* (Campanulaceae), *Verbascum phlomoides* (Scrophulariaceae), and *Rosa arvensis* (Rosaceae). Pollen grain parameters are each categorized by a large gap in the mean values of all species (Table 1) and colour-coded for high (dark) and low (light) values. Pollen collectability is measured by fresh mass of pollen in corbiculae (Fig. 2).

Comparing pollen grain characteristics (Table 1) and their collectability by bumble bees produces a mixed image (Fig. 3). A large pollen grain diameter is no exclusion criterion for collectability as shown by *C*. *pepo*, while spine length is not decisive either as evidenced by *K*. *arvensis*. Low spine density is found among species both with collectable and uncollectable pollen. Even when combining pollen grain characteristics, there is no common denominator that predicts pollen collectability: Pollen grains of the Malvaceae species and *C*. *pepo* share large grains with long spines and low spine density, but differ in collectability (with the limitation that only 40% of tested bumble bees collected *C*. *pepo* pollen).

### Pollen and nectar foragers

**Figure 4:**
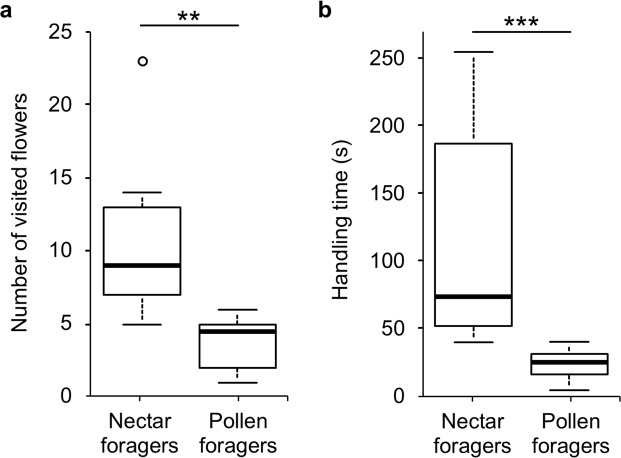
Collection of floral resources by individual *Bombus terrestris* workers from flowers of *Malva sylvestris* (Malvaceae); (**a**) number of visited flowers and (**b**) handling time of flowers for 10 nectar foragers and 10 pollen foragers. Asterisks indicate significance levels after Welch’s *t*-test and Mann-Whitney *U* test respectively (**P = 0.0024; ***P = 0.00028).

On average, nectar foragers visited significantly more flowers than pollen foragers on *M*. *sylvestris* (Fig. 4; Welch’s *t*-test: *t* = 3.948, df = 10.832, *P* = 0.0024) and spent longer handling flowers (on average 86 s; Mann-Whitney *U* test: *U* = 98.5, *P* = 0.0003). Pollen foragers attempted to pack pollen in their corbiculae, but subsequently abandoned their effort and shed the pollen, whereas nectar foragers flew from flower to flower without making any effort to pack the pollen or to groom it off. When released from the net, unsuccessful pollen-collecting bees visited other plant species, whereas successful nectar foragers returned to the nest.

## Discussion

One aim of this study was to link the inability of bumble bees to collect echinate pollen grains with specific pollen properties. Several somewhat divergent hypotheses can be found in the literature: Vansell^35^ deemed the viscid material agglutinating the grains responsible for the incapability of honey bees to gather pollen on cotton flowers (*Gossypium* sp., Malvaceae), Buchmann and Shipman^36^ considered pollen grain size to be the relevant factor. Vaissière and Vinson^26^ have found that honey bees cannot collect pollen of *Abelmoschus esculentus* (Malvaceae) and struggle to pack dried cotton pollen offered in a dish. They exclude pollen grain size and pollenkitt as crucial for packing efficiency and regard the length of the spines as the decisive factor. Lunau *et al*.^27^ have shown that bumble bees are not able to gather natural pollen of *Alcea rosea* (Malvaceae) from artificial flowers and ascribe this to the influence of pollenkitt and spines.

Testing the mechanical defence of echinate pollen grains against collection in a natural setting with unaltered pollen for the first time refutes the conclusions of some previous studies, e.g. concerning the importance of long spines^26,27^. In our study, pollen-foraging bumble bees were incapable of collecting pollen from any of the three Malvaceae species or *Knautia arvensis* (Dipsacaceae). These species share the characteristic of relatively large pollen grains (100–134 µm in diameter) with spines whose length varies greatly. *Lavatera thuringiaca* possesses the longest spines (8.80 µm, 7.9% of the pollen grain diameter) and *K*. *arvensis* the second shortest (1.55 µm, 1.6% of the pollen grain diameter) among all species tested. In contrast, *Cucurbita pepo* pollen was successfully collected by some bumble bees, despite being the largest pollen grains tested with a diameter of 139 µm and spines measuring 5.98 µm (4.3% of the pollen grain diameter). It is known that Cucurbitaceae pollen is collected with varying success by different bee species^37^. Thus, pollen size alone is not a disqualifier for pollen collectability, nor is the echinate exine structure (as shown by the collectability of spiny *C*. *pepo* and *Campanula alliariifolia* pollen). Echinate pollen can be found in several non-related orders of plants, and different exine structures even occur within some genera. Rowley *et al*.^38^ have found that anemophilous Asteraceae species possess pollen with tiny spines. Excluding all pollen properties tested in our experiment from the list of probable causes for uncollectable pollen stated by previous studies leaves only pollenkitt^27,35^. This viscous pollen coating enables adhesion of pollen grains to each other, to flower visitors, and to the stigma, with entomophilous species exhibiting both higher pollenkitt volumes and stronger adhesion than anemophilous species^32^. Consequently, the impact of pollenkitt on pollen packing in corbiculate bees remains to be tested.

Whereas polylectic bumble bees fail to collect echinate pollen of some plant species, oligolectic bee species are often specialized and morphologically adapted to collect such pollen grains. *Ptilothrix plumata* (Emphorini) preferentially forages on the large, spiny pollen grains of *Pavonia* sp. (Malvaceae), which are transported adhering to long hairs on the bees’ tibia^39^. Similar morphological adaptations, i.e. long and sparse hairs, can be found in several genera of bees. For example, the genus *Peponapis* (Eucerini) comprises specialist collectors of Cucurbitaceae pollen^8^, whereas *Andrena hattorfiana* (Andrenidae) is a specialist of a few species of Dipsacaceae^40^. Compared to honey bees and bumble bees that are used for pollination management^35^, native oligolectic bees are often more efficient pollinators of Solanaceae, forage legumes, and Cucurbitaceae^37,41,42^, owing to their visitation frequency (but see King *et al*.^43^; Ballantyne *et al*.^44^) – although these bees effectively deprive the plants of their pollen^45,46^.

In contrast to the scopae of these specialists, the bumble bees tested in our experiments compact pollen in the corbicula, a smooth depression on the tibia of the hind leg framed by rigid bristles. Although the collection of spiny pollen was not entirely inhibited, the eventual compaction of the pollen grains in the corbiculae was not successful when foraging on certain plant species. Owing in part to the size and spines of the pollen grains, which physically impede pollen packing, they presumably interact poorly with the regurgitated nectar used by bees to agglutinate their pollen load. The effort and amount of nectar required to completely envelop large pollen grains (especially with long spines) possibly exceeds the benefit of collecting the pollen, thus resulting in bees discarding the pollen. But as nectar is available in all tested plants with uncollectable pollen, nectar quantity is less likely to be a factor limiting pollen collection. The few individuals that were able to collect the large, echinate pollen of *C*. *pepo* might have been more experienced pollen foragers that had already learned to agglutinate pollen grains more efficiently. However, it is more likely that some other morphological or physiological trait, e. g. pollenkitt, has a species-dependent impact on pollen collectability^27,35^.

Although task specialization in *Apis mellifera* (Apidae) is well-studied (reviewed by Johnson^47^) and the propensity for nectar or pollen foraging has been shown to be connected to the maternal reproductive traits of workers^48^, little is known about the division of foraging tasks in bumble bees. Smith *et al*.^49^ have found that the sensitivity of sensory receptors, size of ovaries, and existing fat reserves do not correlate with foraging task specialization in workers of *B*. *terrestris*. Overall, bumble bee foragers are flexible in the collection of either floral resource^50^ and in adjusting to the colony’s needs regarding the supply of nectar and pollen^51^. We observed that workers focused on collecting either nectar or pollen during a foraging bout (description of the differentiation in methods section). However, pollen foragers also drank some nectar to compensate their energy demand and facilitate pollen collection by agglutinating the grains with regurgitated nectar.

*Malva sylvestris* offers abundant pollen, which covers all flower-visiting bumble bees independently of their foraging task. The echinate structure of Malvaceae pollen results in increased adhesion, thus causing it to strongly adhere to the hair of bees^35,52,53^. While pollen foragers soon ceased trying to collect pollen from *M*. *sylvestris*, they did not – during that foraging bout – shift to collecting nectar from these flowers instead, although they are a lucrative food source (indicated by the fact that nectar foragers readily forage on these flowers). The flowers are not able to train the bumble bees to change their foraging task during one bout, but constitute a rewarding food source for nectar foragers.

To acquire nectar at the base of flowers, foragers often positioned themselves on the column (the fused base of the stamens and pistil), presumably because they could not find a foothold on the smooth epidermal cells of the petals^54,55^. As the most convenient (and energy-efficient) way to gain access to the nectar is by scrambling down the column, the bumble bees regularly contacted the stigmas when approaching the flowers and became covered in pollen after sitting on the anthers. This behaviour enhances the chances of pollination considerably. Gorenflo *et al*.^56^ have found that *Bombus* spp. are very efficient pollinators (based on the number of pollen grains deposited per flower visit), while *A*. *mellifera* is the most effective pollinator (as measured by pollen grains deposited per hour) of *M*. *sylvestris*.

We were able to show that bumble bees cannot collect large pollen grains with an echinate surface structure in three Malvaceae species and *Knautia arvensis* (Dipsacaceae), and that they struggle to collect the similarly shaped pollen of *C*. *pepo*. All uncollectable pollen grains are large and echinate, but not *vice versa* – it is probably (a combination with) another factor, e. g. pollenkitt, that decides collectability of pollen grains for bumble bees. Pollen foragers are almost immediately discouraged by their lack of success, thus minimizing pollen loss caused by pollen-collecting bees. However, bumble bees do not alter their foraging task on the basis of availability of collectable pollen during a single foraging bout. Nectar foragers continue to visit flowers with uncollectable pollen undeterred, thus effectively transferring pollen. The flower constancy of nectar foragers and, more importantly, their negligence of grooming between flower visits makes them ideal pollinators and completely circumvents the pollen dilemma.

## Supplementary information

Original data

## Data Availability

All data generated and analysed during this study are provided as supplementary information (Supplementary Tables S1–4).
