# Supplementary material for: Pollen grain morphology is not exclusively responsible for pollen collectability in bumble bees
Source: Sci Rep. 2019 Mar 18;9:4705. doi: 10.1038/s41598-019-41262-6 (PMC6423004; doi:10.1038/s41598-019-41262-6)
Supplement: Supplementary file 1 — Original data [file 41598_2019_41262_MOESM1_ESM.pdf]

Supplementary Information

Pollen grain morphology is not exclusively responsible for pollen collectability in bumble bees

Sabine Konzmann*, Sebastian Koethe, Klaus Lunau

Institute of Sensory Ecology, Heinrich Heine University Düsseldorf, Germany
*sabine.konzmann@uni-duesseldorf.de

Original data

Supplementary Table S1: Pollen grain diameter [µm] of all plant species tested in the experiment.

Alcea ficifolia	*Lavatera thuringiaca*	*Malva sylvestris*	*Knautia arvensis*	*Cucurbita pepo*	*Campanula alliariifolia*	*Verbascum phlomoides*	*Rosa arvensis*
130.86	111.09	101.90	100.21	146.31	33.94	24.43	30.94
127.32	109.04	89.63	99.89	142.47	36.00	22.74	29.49
136.51	108.98	100.67	104.38	144.49	34.70	23.15	28.97
133.60	116.09	108.66	103.35	136.75	36.98	21.93	26.34
127.38	113.57	100.69	96.42	144.94	30.25	23.16	30.25
135.39	105.38	99.92	97.58	133.60	31.48	22.31	29.63
141.96	111.81	106.79	102.50	148.10	28.35	20.75	30.83
134.56	113.50	106.81	93.39	97.86	36.17	22.13	28.11
143.33	108.49	110.01	96.66	139.82	39.31	21.13	32.76
130.11	112.32	116.63	103.27	159.82	32.96	23.07	28.51

Supplementary Table S2: Pollen spine number [n] of all tested plant species with echinate pollen grains.

Alcea ficifolia	*Lavatera thuringiaca*	*Malva sylvestris*	*Knautia arvensis*	*Cucurbita pepo*	*Campanula alliariifolia*
176	120	128	112	80	152
168	120	128	88	88	200
184	128	136	152	120	168
272	120	128	152	112	208
248	128	136	136	136	216
216	144	144	144	152	160

Supplementary Table S3: Pollen spine length [µm] of all tested plant species with echinate pollen grains.

Alcea ficifolia	*Lavatera thuringiaca*	*Malva sylvestris*	*Knautia arvensis*	*Cucurbita pepo*	*Campanula alliariifolia*
9.96	9.06	6.51	1.92	5.00	1.10
10.05	7.42	6.52	1.34	5.46	0.90
12.82	8.53	5.23	1.50	4.85	0.90
10.86	7.36	7.16	1.27	4.85	0.90
11.27	6.75	6.32	1.82	4.27	0.80
9.70	8.55	7.08	1.23	5.25	0.90
10.50	8.85	7.67	2.16	5.22	1.00
10.74	7.98	5.79	2.18	5.06	1.10
10.13	8.53	8.38	1.61	4.12	1.10
10.74	8.79	5.96	1.89	4.61	0.90
10.29	9.70	6.76	1.69	5.23	
9.56	10.50	8.15	1.61	4.60	
9.75	8.79	7.89	1.08	5.84	
8.83	8.59	4.13	1.61	4.95	
7.43	11.06	6.76	1.50	5.82	
13.46	9.84	7.00	1.50	6.14	
10.22	9.37	5.00	2.01	5.90	
10.16	8.79	6.81	1.34	5.86	
12.37	7.49	5.28	1.08	5.66	
10.90	8.68	7.67	1.69	4.95	
9.63	6.86	7.06	1.27	6.21	
12.38	9.06	7.29	1.75	7.00	
9.45	8.68	6.73	1.89	5.84	
8.92	8.55	5.23	1.50	4.77	
9.88	10.72	5.14	1.20	6.52	
9.59	10.12	6.45	1.53	5.90	
7.98	7.76	6.94	1.34	5.64	
8.19	8.35	8.05	0.85	5.43	
11.00	7.39	5.66	1.53	4.96	
8.66	8.59	6.44	1.61	6.18	
8.64	9.29	6.61	1.53	8.19	
9.90	11.11	6.96	1.92	7.47	
7.52	7.98	5.71	2.01	7.39	
7.89	8.07	5.53	2.39	6.26	
10.58	7.49	7.00	1.92	7.87	
11.24	9.12	8.26	1.23	6.47	
10.64	9.99	8.24	1.50	7.22	
9.75	10.74	7.49	1.23	6.96	
9.35	11.06	7.16	1.08	6.45	
11.50	8.61	7.00	1.92	6.50	
10.67	7.86	7.67	1.56	7.08	
12.81	8.23	7.62	1.69	6.50	
12.06	9.70	5.44	1.56	6.73	
8.96	7.47	7.58	0.84	6.21	
13.11	7.39	8.19	0.99	5.66	
10.09	10.21	6.79	1.89	7.00	
9.82	9.39	7.03	1.29	6.94	
9.07	7.49	7.37	1.10	7.67	
9.45	9.53	7.69	1.96	5.93	
10.43	8.61	6.07	1.53	6.45	

Supplementary Table S4: Original data of the pollen collectability and nectar foraging tests.

Plant species	Handling time [s]	Visited flowers [n]	Pollen mass [mg]
Alcea ficifolia	15.3	2	0
Alcea ficifolia	11.6	1	0
Alcea ficifolia	13.6	2	0
Alcea ficifolia	27.2	2	0
Alcea ficifolia	9.2	1	0
Alcea ficifolia	21	1	0
Alcea ficifolia	14.2	3	0
Alcea ficifolia	16	2	0
Alcea ficifolia	7.6	1	0
Alcea ficifolia	11.2	1	0
Lavatera thuringiaca	13.2	3	0
Lavatera thuringiaca	10.0	3	0
Lavatera thuringiaca	20.8	5	0
Lavatera thuringiaca	12.6	3	0
Lavatera thuringiaca	4.7	2	0
Lavatera thuringiaca	5.7	1	0
Lavatera thuringiaca	7.4	2	0
Lavatera thuringiaca	18.2	3	0
Lavatera thuringiaca	12.3	3	0
Lavatera thuringiaca	25.4	5	0
Malva sylvestris	30.9	5	0
Malva sylvestris	23.7	2	0
Malva sylvestris	23.7	4	0
Malva sylvestris	14.5	2	0
Malva sylvestris	39.7	5	0
Malva sylvestris	16.5	4	0
Malva sylvestris	27.1	5	0
Malva sylvestris	40.5	6	0
Malva sylvestris	4.4	1	0
Malva sylvestris	30.5	5	0
Malva sylvestris	52.0	12	nectar forager
Malva sylvestris	186.8	23	nectar forager
Malva sylvestris	47.9	9	nectar forager
Malva sylvestris	247.5	13	nectar forager
Malva sylvestris	58.3	5	nectar forager
Malva sylvestris	76.5	14	nectar forager
Malva sylvestris	70.2	7	nectar forager
Malva sylvestris	39.7	9	nectar forager
Malva sylvestris	79.3	7	nectar forager
Malva sylvestris	254.5	8	nectar forager
Knautia arvensis	22.6	7	0
Knautia arvensis	17	6	0

Knautia arvensis	29.3	6	0
Knautia arvensis	13.5	4	0
Knautia arvensis	12.5	1	0
Knautia arvensis	145.4	12	0
Knautia arvensis	47.8	6	0
Knautia arvensis	153.3	17	0
Knautia arvensis	24	3	0
Knautia arvensis	23.7	4	0
Cucurbita pepo	193.4	6	0.6
Cucurbita pepo	146.9	4	0
Cucurbita pepo	183.2	6	0
Cucurbita pepo	223.0	5	0
Cucurbita pepo	166.0	4	0
Cucurbita pepo	231.2	3	0.3
Cucurbita pepo	79.5	2	0
Cucurbita pepo	157.6	3	13.2
Cucurbita pepo	102.3	4	0
Cucurbita pepo	133.1	6	13.7
Campanula alliariifolia	194.4	14	13.4
Campanula alliariifolia	268	8	0.3
Campanula alliariifolia	261.4	9	3.7
Campanula alliariifolia	226.3	20	12.5
Campanula alliariifolia	206.6	11	14.3
Verbascum phlomoides	164.1	40	21.2
Verbascum phlomoides	122.5	14	1.2
Verbascum phlomoides	153.4	21	13.5
Verbascum phlomoides	104.9	14	3.9
Verbascum phlomoides	38.1	6	1.33
Rosa arvensis	174.3	17	13.6
Rosa arvensis	208.7	9	5.6
Rosa arvensis	128.3	14	2
Rosa arvensis	215.9	14	8.3
Rosa arvensis	216.7	17	18.5
